# The role of virtual reality as adjunctive therapy to spinal cord stimulation in chronic pain: A feasible concept?

**DOI:** 10.3389/fpain.2023.1094125

**Published:** 2023-02-27

**Authors:** Timothy Noble, Lyndon Boone, Antonios El Helou

**Affiliations:** ^1^ Faculty of Medicine, Memorial University of Newfoundland, St. John's, NL, Canada; ^2^ Division of Neurosurgery, Horizon Health Network, Moncton, NB, Canada

**Keywords:** virtual reality, spinal cord stimulation (SCS), chronic pain, neuromodulation, cognitive behavioral therapy (CBT)

## Abstract

Spinal cord stimulation and virtual reality therapy are established and promising techniques, respectively, for managing chronic pain, each with its unique advantages and challenges. While each therapy has been the subject of significant research interest, the prospect of combining the two modalities to offer a synergistic effect in chronic pain therapy is still in its infancy. In this narrative review, we assess the state of the field combining virtual reality as an adjunctive therapy to spinal cord stimulation in chronic pain. We also review the broader field of virtual reality therapy for acute and chronic pain, considering evidence related to feasibility in the Canadian healthcare system from cost and patient satisfaction perspectives. While early results show promise, there are unexplored aspects of spinal cord stimulation combined with virtual reality therapy, particularly long-term effects on analgesia, anxiolysis, and implications on the effectiveness and longevity of spinal cord stimulation. The infrastructure for billing virtual reality as a consult service or therapy must also catch up if it is eventually used to supplement spinal cord stimulation for chronic pain.

## Introduction

It is difficult to overestimate the societal impact of chronic pain, defined as an aversive sensory or emotional experience associated with actual or potential tissue injury recurrent for longer than three months (1, 2). While acute pain carries survival value as a signal of physical injury or illness, chronic pain, in itself, can be categorized as a disease rather than a symptom, carrying significant long-term treatment implications for patients and healthcare providers (3, 4). It is estimated that one in five Canadians lives with chronic pain. The direct (health care) and indirect (lost production) cost of chronic pain in Canada was $38.2–$40.3 billion in 2019, with the direct cost representing over 10% of the total combined Canadian health expenditure (5). Until recently, opioids were typically used to treat chronic pain; however, the chronic use of opioids can produce side effects such as dependence and increased occurrence of addiction, and misuse has driven the importance of non-pharmacological long-term treatment options for chronic pain (6, 7).

Spinal cord stimulation (SCS) has emerged as an alternative treatment modality for chronic pain—in particular, failed back surgery syndrome, complex regional pain syndrome, and peripheral neuropathies—when conventional (e.g., pharmacological) therapies produce insufficient pain relief or adverse effects (8, 9). While the mechanisms behind SCS are complex and imperfectly understood, it is believed that stimulation to the dorsal spinal cord activates fast-transmitting Aβ fibres and hence inhibitory interneurons, which block the transmission of pain signals through the spinal cord, thus “closing the gate” to perceived pain (10). Randomized control trials and meta-analyses have consistently demonstrated improved pain scores and quality of life in chronic pain patients with SCS compared to sham/placebo stimulation or no SCS (11, 12).

During the past two decades, virtual reality (VR) technologies have become an increasingly common non-pharmacological therapy to deliver or support pain-related treatments and have been shown to produce analgesic effects in both acute and chronic pain settings (13, 14). VR is an artificial computer-based environment designed to create real-world sensory inputs using distinct combinations of interaction devices and sensory display systems (14, 15). VR therapy for pain relief indicates attentional processes, namely distraction, as the primary mechanism for the analgesic effects of VR (15–17). VR-induced distraction involves diverting an individual's finite attentional resources away from the perception of pain toward other bodily sensations, thereby reducing the cognitive capacity to process painful stimuli and diminishing the experience of pain (14, 15, 18). Compared to traditional methods of distraction, VR is theorized to be more effective due to its immersive nature, as VR can avail of a concurrent combination of visual, auditory, and tactile stimuli that, in theory, command more attentional resources and provide more effective pain relief (14, 18). To achieve adequate pain relief, distraction must engage essential cognitive resources to positive stimuli incongruent with pain (17). More immersive VR environments can elicit greater cognitive resources, increasing engagement and presence in the virtual environment, thus decreasing attentional processes for pain sensation and the experience of pain itself (17–19). Although the theory of VR-induced distraction supports decreased pain during VR treatment in acute pain conditions, the active and continuous use of VR to treat chronic pain is unrealistic, thus investigating the mechanisms by which VR could induce long-term analgesic effects is integral to extending the use of VR beyond the acute setting (20).

Despite research supporting the analgesic potential of both VR and SCS in treating chronic pain, current treatment options are not without limitations. Notably, most longitudinal studies indicate that the lifetime of pain relief from SCS is finite, with a loss of therapeutic effect occurring over several years (21–23). Due to the promise of each treatment for chronic pain, the original intention of the paper was to conduct a systematic review of the efficacy of VR as an adjunctive therapy to SCS for chronic pain management. On October 13, 2022, an Ovid MEDLINE search of relevant terms (Supplementary Table S1) yielded 20 results. After two authors screened each title and abstract, only one study was found to investigate a combined SCS-VR approach in chronic pain. Due to the limited body of literature regarding concurrent VR therapy and SCS, we instead turned our focus to the feasibility of VR as an adjunctive therapy to SCS in chronic pain. Here we discuss existing literature on concurrent VR-SCS therapy and a brief review of the evidence for the effectiveness of VR as an analgesic in other contexts. Finally, we summarize findings on the feasibility of VR therapy concomitant with SCS.

## Virtual reality as an analgesic in acute and chronic pain

VR experiences can be categorized into two modalities, immersive and non-immersive. Non-immersive VR allows the user to maintain limited sensory (e.g., visual) connections with the real-world environment. Conversely, immersive VR uses specialized equipment such as head-mounted displays (HMDs) to increase the integration of multi-sensory experiences and replace the users' real-world surroundings with a virtual environment (18, 24). With consumer HMDs becoming more affordable, research exploring VR as an analgesic has increasingly moved towards immersive, HMD-based VR systems (16, 25, 26). Some studies indicate that immersive VR therapy using HMDs may be more effective for pain relief than non-immersive VR (17, 19). While many consumer-grade HMDs have been used in therapeutic research [see Summary Tables in (16, 25, 26) for examples], some companies now offer HMD products and plans geared towards comprehensive services and support^1^. VR activities used during treatment are numerous and, to some degree, dependent on the context and setting of treatment. Common activities include purchasable and custom VR games, meditation and mindfulness activities, and viewings of movies, videos, and vivid scenery (16, 25, 26).

The ability of VR therapy to acutely reduce pain has been well-established in various contexts, including during procedures, post-surgical recovery, cancer pain, palliative care, and among patients suffering from chronic pain (25–28). Most reviewed studies use pain intensity, commonly measured by a visual analog scale (VAS) or a numeric rating scale (NRS), as the primary outcome. Systematic reviews and meta-analyses have shown that the majority of studies demonstrate a statistically significant improvement in pain reduction during and shortly after VR therapy, as measured by these outcomes (25, 26). In a study investigating the use of VR therapy to reduce chronic pain in patients with knee osteoarthritis, Sarkar and colleagues even suggest that moderate analgesic effects could last into the next day following VR therapy (29). The heterogeneity across studies in patient populations assessed, conditions treated, and timeframes/contexts of VR treatment administration and pain scoring do, however, make the pooling of results and analyses difficult. Differences in effectiveness between these contexts should become clear as researchers conduct more studies involving VR as an analgesic.

The field of VR as a treatment for chronic pain is still in its developmental stages, meaning that multiple components of VR interventions for chronic pain are hypervariable, and experimental findings should be interpreted with caution. Accordingly, comprehensive assessment of VR interventions for chronic pain poses a challenge. A systematic review conducted by Mallari and colleagues evaluated the effectiveness of VR in reducing chronic pain (25). Analysis of the studies suggested that VR has the potential to reduce pain levels in patients with chronic pain during VR use, however inconsistent findings limited the viability of VR to reduce pain after exposure (24), concluding that VR is unlikely to produce lasting analgesic effects for chronic pain due to current treatment protocols and inconsistent VR intervention methodology. Further, the complex nature of chronic pain and associated neuroplastic mechanisms, VR methods used in chronic pain relief are incongruent in the type of VR hardware and software (16). A recent review on the analgesic effects of VR for people with chronic pain reported that VR applications ranged from exercise to cognitive behavioural therapy (e.g., embodiment), distraction, and virtual limb movement (16). Further, studies varied in VR duration, session frequency, and additional concurrent interventions, leading to a discrepancy in an individual's experience in VR (16). Inconsistency in VR methodology parallels past findings (14, 25), which may limit the perceived efficacy of VR in chronic pain relief. Despite this, research has shown that more frequent VR treatments that occur over a longer duration showed greater pain reduction (25) and even continual analgesic effects (16). Overall, past research has expressed cautious support for the analgesic effects of VR in chronic pain (14, 17, 18, 20, 25); however, further and more standardized research is required.

## Combining analgesic modalities: virtual reality and spinal cord stimulation

To date, the only study we found that directly investigates a combined SCS-VR approach is that by Solcà and colleagues, who sought to address the diminishing of SCS-induced analgesia over time by enhancing SCS with concomitant VR therapy (30). They designed a custom, immersive VR application that allows the patient to see a model of their own body integrated into a 360° stereoscopic video environment as if seen from the first-person perspective. With and without concomitant SCS, chronic leg pain patients viewed this model of their own legs through VR, with visual feedback (a highlighted region) superimposed on top of the leg region where they typically felt paresthesia from SCS. They found that this VR therapy, concomitant with SCS, significantly reduced pain ratings during and 10 min after VR therapy compared to VR therapy alone. Solcà and colleagues made the connection that concomitant SCS-VR induced changes in leg embodiment, with patients feeling that the artificial illumination from the VR system was causing their tingling (paresthesia) sensation. Riva and colleagues previously identified the inability to stimulate the internal body as a critical limiting factor in the therapeutic potential of VR therapy (31), and recently proposed the concept of sonoception to address this limitation (32). Thus, the integrated SCS-VR therapy proposed by Solcà and colleagues could act in a synergistic manner, addressing therapeutic shortcomings in both strategies in order to produce enhanced analgesic SCS for chronic pain with personalized VR. Similarly, studies using a combination of VR and transcranial direct current simulation (tDCS), compared to tDCS alone produced enhanced synergistic effects, resulting in an extended duration of analgesic effects (33). Soler and colleagues also demonstrated that transcranial stimulation (9) combined with visual illusion (34), a non-immersive VR technique, reduced the intensity of neuropathic pain significantly more than either of the single interventions with persistent effects lasting up to 12 weeks after treatment (35). These studies further support the potential of concurrent VR-neuromodulatory techniques in pain reduction.

## Psychological and behavioural benefits of virtual reality

Psychological factors, including attitude and emotion, play a critical role in an individual's experience of pain (36). Cognitive behavioural therapy (CBT) is a psychological treatment for chronic pain that reduces psychological distress by decreasing maladaptive behaviours, increasing adaptive behaviours, and identifying and correcting maladaptive thoughts and beliefs (36, 37). Chronic pain is associated with psychological disorders such as depression and anxiety, which can exacerbate the effects of each condition (36). At the same time, positive emotions are proposed to reduce perceived pain (38). VR is suggested to alleviate both depression and anxiety in chronic pain (39) while also producing improvements in positive affect (40). Of note, VR exposure therapy for anxiety (specifically, phobias) was reported to be significantly more effective than traditional exposure (the gold standard in the field) (41). Although the underlying psychological and behavioural principles in VR treatment for chronic pain are similar to those in CBT, the versatility of VR indicates a therapeutic edge over conventional cognitive and behavioural therapies. In many chronic pain conditions, physical touch or movement is often counterproductive as it may increase or induce pain (30). Conversely, VR can enhance observational learning, subsequently providing a positive response to induce a long-lasting change in behaviour due to positive reinforcement and disproval of previously held maladaptive conceptions about one's body (42). Further, recommendations for the treatment of chronic pain include combining pharmacological, physical, and psychological interventions with multicomponent psychological interventions suggested to be more useful (40). One example of the multicomponent approach is VR and transcranial direct current simulation (tDCS) in spinal cord injury, where VR visual illusion of the body combined with tDCS produced synergistic effects of the intervention and more prolonged analgesic effects (33). Recently, Botella and colleagues designed a multicomponent cognitive-behavioural program using VR to deliver relaxation and mindfulness in a chronic pain condition (fibromyalgia), with the treatment producing long-term physical and psychological benefits in fibromyalgia patients (40).

## Patient perception of virtual reality

Recent studies generally report positive reception to virtual reality therapy from patients. Most middle-aged and older patients find VR headsets comfortable to use, and patients with glasses can wear the headsets over their glasses without discomfort (29, 43, 44). Some of the complaints described by a minority of patients include feeling uncomfortable and claustrophobic, concerns about the heaviness of the headset, a desire for more realistic scenes in mediation-based programming, and concerns that VR does not feel like “real” treatment compared to alternative manual treatments (29, 43, 45). Deo and colleagues describe that while some patients appreciate how the VR headset blocked their sight of doctors and equipment, which they felt to be particularly anxiety-provoking during procedures, others preferred to be more aware and to be able to talk with the doctor. It should be re-emphasized, however, that the majority of patients in the studies we reviewed exhibited an overall positive experience with VR therapy, and these complaints appear to be incident in the minority of cases.

## Cost considerations for virtual reality therapy adjunctive to spinal cord stimulation

Research investigating the cost-analysis of implementing VR therapy for chronic conditions proposed an annual cost of $7544 USD for the VR system hardware, software and technical support (46). As compared to conventional therapy for chronic conditions, VR implementation becomes most cost-effective when more patients take part in the program (46). Although SCS produces effective analgesic effects for chronic pain conditions, SCS is susceptible to high rates of complications (e.g., device removal or loss of therapeutic effectiveness), severely impacting overall efficacy and healthcare costs (23). Concurrent VR-SCS therapy for chronic pain is theorized to act in a synergistic fashion, increasing the efficacy for chain pain management, as well as overall cost-effectiveness. Similar to VR-SCS, Xi and colleagues (2021) found that the combination of virtual illusion (VI) and tDCS for spinal cord injury resulted in greater and more sustained reductions in pain levels, which in turn produced economic benefits (47). Within three months of intervention, the initial cost of VI-tDCS therapy was virtually offset by reductions in health care costs due to improvements in condition severity. Further, by one year post treatment cumulative costs for both VI and tDCS treatment were lower than that of standard care. Although not directly comparable to VR-SCS therapy, the underlying premise of providing more effective, long-lasting analgesic effects can produce beneficial financial outcomes in the long term. Moreover, Leemhuis and colleagues observed similar effects with VR visual illusion combined with tDCS producing synergistic results and longer analgesic effects (33).

## Discussion

While SCS has proven to be an effective treatment for chronic pain, the therapeutic effects typically diminish with time, highlighting long-term treatment-limiting complications of SCS (21–23). Meanwhile, immersive VR has emerged as a contemporary analgesic and distraction from pain with proven benefits in the acute timeframe but limited evidence of long-term pain reduction for chronic pain. Given the acute nature of VR analgesia, some researchers have suggested that VR could be best suited for relief from transient pain experiences. More significant reductions in pain intensity over extended periods may be best achieved through multimodal treatment approaches (16, 25). While attempts at combining SCS and VR have been limited, the two could exhibit a synergistic effect in long-term, chronic settings based on their distinct advantages and mechanisms. Such a synergistic effect could be hypothesized to have several implications for SCS effectiveness and chronic pain management in the long run, including lower pain intensity scores, extended battery life for SCS stimulators due to decreased dependence on stimulation, and extended windows for therapeutic effect.

To our knowledge, the only work to date combining immersive VR with SCS is the work by Solcà and colleagues, discussed in Section 3 (30). While their results are promising for the potential synergy between VR and SCS, the authors acknowledged multiple biases that limit the conclusions that can be drawn from their work. First, their test conditions (i.e., congruent SCS-VR vs. VR alone) were easily distinguishable due to paresthesia induced by SCS in the region of pain, limiting their ability to randomize experimental conditions fully. Second, congruent SCS-VR was not compared to an SCS-alone condition to demonstrate bidirectional synergistic improvement. As such, while Solcà and colleagues have provided a foundation to build on, we believe that more work is needed to adequately demonstrate the potential of SCS in enhancing the analgesic properties of VR and vice versa. The first limitation described regarding their work may be overcome in future studies by implementing SCS using either high-frequency (10-kHz) or “burst” stimulation, which have demonstrated pain relief—in some cases greater than that of conventional SCS—in the absence of paresthesia (48–50). Importantly, we believe that to investigate the full potential benefits of concomitant SCS and VR, researchers should look to assess the long-term implications and impacts of combined SCS and VR therapy, including effects on sustained pain reduction, stimulator health and battery life, and longevity of therapeutic effect.

Studies investigating ease-of-use and patient reception to VR have largely focused on acute settings (e.g., inpatient or outpatient clinic) with trained staff or research personnel available to assist with VR operations and administration. While studies offer strong support for ease-of-use and positive patient experience under such conditions, a more mature model of chronic therapy could be speculated to involve prolonged and frequent VR usage at home with patients having their own headset. It is unclear whether ease-of-use scores and patient satisfaction—supposed prerequisites for long-term treatment feasibility—will remain high in long-term, at-home settings, particularly for older patients. Side effects from VR use could also be more difficult to monitor and practice in these settings. While some therapy-oriented VR companies offer comprehensive packages with monthly or weekly guided sessions or online support facilitating ease-of-use, these options are expensive, and could be less feasible compared to self-administered, at-home VR for patients in a Canadian healthcare system where billing practices and insurance policies for VR are in their infancy compared to the US. As such, economies that are strained for healthcare resources could benefit from more studies that explore the feasibility of self-administered, at-home VR therapy.

Meanwhile, billing practices and codes for virtual reality therapy are still in development in North America, with VR therapy codes proposed by the American Medical Association for 2023 (51, 52). While companies in the US have found ways to offer VR therapy services covered through health insurance, we were unable to find evidence for infrastructure in Canada to financially support VR as a consult service or therapy. Until policies and billing practices are established, VR therapy in Canada may be confined to inpatient and clinic settings, or research trials. In the meantime, there appears to be plenty of avenues for research to further explore and solidify the value of VR therapy, particularly in chronic pain management, at-home settings, and as an adjunctive therapy to complimentary therapy modalities such as SCS. The positive results demonstrated thus far for VR as an analgesic and anxiolytic suggest, however, that the demand for VR therapy may not be far away. As such, the exploration of cost models and billing practices for providers may be a worthwhile pursuit even in these early stages of VR therapy in Canada.

In summary, VR has shown promising effects for short-term reductions in chronic pain although combination with SCS may provide a better long-term pain control, and a reduction in flare-ups which could reduce emergency visits. If VR has the advantage of providing a continuous cognitive-behavioural component on top of physical electrical, this will provide a better and more prolonged pain control in chronic pain patients.

## Author contributions

TN and LB: contributed equally to the literature review and writing of the first draft manuscript. TN, LB, and AEH: contributed equally to the initial conception of the study and its methodology. All authors contributed to the article and approved the submitted version.

## Footnotes

^1^https://www.xr.health/; https://www.appliedvr.io/
