# Supplementary material for: The role of virtual reality as adjunctive therapy to spinal cord stimulation in chronic pain: A feasible concept?
Source: Front Pain Res (Lausanne). 2023 Feb 27;4:1094125. doi: 10.3389/fpain.2023.1094125 (PMC10009231; doi:10.3389/fpain.2023.1094125)
Supplement: Supplementary file 1 [file Table1.docx]

Supplementary Material

The role of virtual reality as adjunctive therapy to spinal cord stimulation in chronic pain: a feasible concept?

Timothy Noble^1†^, Lyndon Boone^1†^, Antonios El Helou^1,2*^

^1^Memorial University of Newfoundland, Faculty of Medicine, St. John’s, NL, Canada

^2^Horizon Health Network, Division of Neurosurgery, Moncton, NB, Canada

†These authors contributed equally to this work and share first authorship

*** Correspondence:**

Dr. Antonios El Helou
Dr.Antonios.Elhelou@horizonnb.ca

# Literature search strategy

On October 13, 2022, we executed the following search in Ovid MEDLINE, yielding 20 results in total which were screened (title and abstract) by two authors for the use of both virtual reality (VR) and spinal cord stimulation (SCS) in chronic pain (Table S1).

**Table S1**: Search strategy for papers combining VR with SCS in chronic pain. Individual queries are listed in the middle column, indexed by the leftmost column. The rightmost column quantifies the results returned from Ovid MEDLINE from each query.

| **Index** | **Search terms** | **Results** |
| --- | --- | --- |
| 1 | ((chronic* or nonacute* or non-acute* or subacute* or sub-acute* or persist* or long* or consist* or recur*) adj3 pain*).kf,tw. | 108403 |
| 2 | Chronic Pain | 20755 |
| 3 | Complex Regional Pain Syndromes/ | 1780 |
| 4 | (complex adj3 pain adj2 syndrome*).kf,tw. | 3682 |
| 5 | 1 or 2 or 3 or 4 | 113893 |
| 6 | ((virtual* or augment* or mixed or extend*) adj3 (realit* or virtualit*)).kf,tw. | 18680 |
| 7 | computer simulation/ or augmented reality/ or virtual reality/ | 212010 |
| 8 | (oculus rift or oculusrift or reverb or hololens or holoanatomy or infolinker).tw,kf. | 422 |
| 9 | (virtual adj4 (event* or train* or course* or curricul* or learn* or scenario* or simulat* or environment* or object* or class* or project* or educat* or opportunit*)).kf,tw | 17591 |
| 10 | (simulat* adj4 (environment* or object* or event* or scenario*)).tw,kf. | 22189 |
| 11 | (computer adj1 generat* adj1 (environment* or object* or event* or scenario* or simulation)).tw,kf | 82 |
| 12 | Simulation Training/ | 5705 |
| 13 | 6 or 7 or 8 or 9 or 10 or 11 or | 253914 |
| 14 | 5 and 13 | 410 |
| 15 | (spin* adj2 cord adj2 (neuromodulat* or stimulat*)).kf,tw. | 4620 |
| 16 | Spinal Cord Stimulation/ | 1637 |
| 17 | 15 or 16 | 4857 |
| 18 | 5 and 13 and 17 | 20 |
